# Incubation temperature alters stripe formation and head colouration in American alligator hatchlings and is unaffected by estradiol-induced sex reversal

**DOI:** 10.1242/jeb.245219

**Published:** 2023-03-31

**Authors:** Grace Rogerson, Samantha Bock, Yeraldi Loera, Benjamin Parrott, John F. Mulley

**Affiliations:** ^1^ School of Natural Sciences, Bangor University, Deiniol Road, Bangor, Gwynedd LL57 2UW, UK; ^2^ Odum School of Ecology, University of Georgia, Athens, GA 30602, USA; ^3^ Savannah River Ecology Laboratory, University of Georgia, Aiken, SC 29802, USA; ^4^ Department of Ecology & Evolutionary Biology, Princeton University, Princeton, NJ 08544, USA

**Keywords:** Stripe formation, Incubation temperature, Alligators, Climate change, Sex reversal, Temperature-dependent sex determination

## Abstract

Considerations of the impact climate change has on reptiles are typically focused on habitat change or loss, range shifts and skewed sex ratios in species with temperature-dependent sex determination. Here, we show that incubation temperature alters stripe number and head colouration of hatchling American alligators (*Alligator mississippiensis*). Animals incubated at higher temperatures (33.5°C) had, on average, one more stripe than those at lower temperatures (29.5°C), and also had significantly lighter heads. These patterns were not affected by estradiol-induced sex reversal, suggesting independence from hatchling sex. Therefore, increases in nest temperatures as a result of climate change have the potential to alter pigmentation patterning, which may have implications for offspring fitness.

## Teaser

**Summary:** Incubation temperature alters the number of stripes on the dorsal surface of alligator hatchlings and changes head pigmentation, with potential implications for hatchling camouflage and thermoregulation.

## INTRODUCTION

Over a fifth (21.1%) of reptile species are classified as ‘Vulnerable’, ‘Endangered’ or ‘Critically Endangered’ on the International Union for Conservation of Nature (IUCN) Red List (Cox et al., 2022). Threats include habitat loss and change, disease, the impact of introduced species, and human activities such as pollution and hunting (Gibbons et al., 2000; Böhm et al., 2013; Cox et al., 2022). The impact of climate change on reptiles is multifaceted, and likely to result in habitat fragmentation, range shifts, telomere shortening and skewed sex ratios in species with temperature-dependent sex determination (Roberts, 1988; Gibbons et al., 2000; Ryberg and Lawing, 2018; Bae et al., 2022; Cox et al., 2022). Indeed, nest sites of several species are already producing highly skewed or entirely single-sex broods (Jensen et al., 2018; Bock et al., 2020). However, one aspect of reptilian biology that has not previously been considered in the context of climate change is pigmentation patterning.

Reptiles exhibit a wide diversity of pigment patterns, from longitudinal and lateral stripes to spots and complex reticulated patterns, all of which result from the distribution of pigment-producing cells in the skin. Vertebrate pigment cells are neural crest derivatives, and their ultimate location is determined by the interplay of multiple signalling pathways during embryonic development (Kelsh et al., 2009; Bronner and LeDouarin, 2012). There is an extensive body of work on the formation of animal pigment patterns, mostly deriving from major model-based contributions by Alan Turing on the chemical basis of morphogenesis (Turing, 1952), and Lewis Wolpert on positional information (Wolpert, 1969). Both models involve diffusible molecular components, and, as rates of diffusion are impacted by temperature changes, altered incubation temperatures might influence pigmentation patterning. Increased incubation temperature can also affect embryonic growth and time to hatching in reptiles (Deeming and Ferguson, 1989; Du and Ji, 2003; Dormer et al., 2016; Tezak et al., 2020), influencing the area over which these processes are active. Such size-dependent pattern formation has previously been proposed for the formation of stripes on American alligator (*Alligator mississippiensis*) hatchlings, where those incubated at higher (male-promoting) temperatures have on average one more stripe than those incubated at lower (female-promoting) temperatures, and those incubated at intermediate temperatures have an intermediate phenotype (Murray et al., 1990). These experiments utilized a relatively small sample size (31 eggs per temperature) and differentiated temperature effects from a sex effect using a ‘shift twice’ system during the thermosensitive stage of sexual development, where eggs to be incubated at 33°C until hatching experienced a period at 30°C between days 7 and 14 (Deeming and Ferguson, 1988; Murray et al., 1990). At least some individuals in that study therefore experienced two different incubation temperatures, and recent research has shown that the temperature-sensitive window may be wider than previously thought (McCoy et al., 2015).

We therefore set out to investigate the effect of incubation temperature on alligator pigmentation patterning using both a larger sample size and hormone-induced sex reversal, where *in ovo* exposure to 17β-estradiol (E2) feminizes embryos incubated at what should be male-producing temperatures (Bull et al., 1988; Kohno et al., 2015). If stripe formation is indeed influenced only by incubation temperature and not sex, animals incubated at higher temperatures with E2 treatment should show a higher ‘male’ stripe number, even though they are phenotypically female. We also sought to investigate non-stripe-related pigmentation changes, namely differential head colouration in hatchlings, which anecdotal evidence from our previous experiments suggested might vary with incubation temperature.

## MATERIALS AND METHODS

### Animals

Eight clutches of alligator [*Alligator mississippiensis* (Daudin 1802)] eggs (*N*=372) were collected from wild nests within 48 h of oviposition at the Tom Yawkey Wildlife Center (Georgetown, South Carolina) under Department of Natural Resources permit SC-08-2019, and transported to the Savannah River Ecology Laboratory (Aiken, South Carolina). All experimental procedures were approved by the University of Georgia's Institutional Animal Care and Use Committee (protocol A2019 06-001-R1). Eggs were candled to determine viability (Ferguson, 1985), transferred to damp sphagnum moss, and kept at a constant 32°C. At developmental stage 15, the earliest stage at which alligator sex determination responds to temperature (McCoy et al., 2015), eggs were randomly assigned to either a constant male-promoting temperature (33.5°C) or a constant female-promoting temperature (29.5°C). We predicted the date of oviposition for each clutch by staging one representative embryo (Ferguson, 1985) from each clutch and, using the established relationship between developmental rate and temperature (Kohno and Guillette, 2013), predicted the date at which each clutch would reach stage 15. At developmental stage 19, eggs were topically dosed with either a vehicle control (0.5 ml g^−1^ egg mass absolute ethanol) or E2 (0.5 μg g^−1^ egg mass; Sigma-Aldrich) at a dose previously shown to induce complete sex reversal at a male-promoting temperature (Kohno et al., 2015). It takes embryos 11 days to develop from stage 15 to stage 19 at 29.5°C, and 9 days at 33.5°C, and so embryos incubated at the higher temperature received the topical hormone/vehicle treatment 2 days earlier than their clutch mates at the lower temperature. Eggs were then returned to their respective temperature conditions and incubated until hatching. Hatchlings were photographed using a standard-mounted digital camera (Canon EOS M50 with EF-N15-45 mm f/3.5-6.3 IS STM lens) positioned above the animal on a gridded mat (Zerro A4 cutting mat, 9×12 inches) with a reference colour standard (24ColorCard, CameraTrax) and no flash under consistent lighting.

### Stripe counts and head colouration

Individual photographs were imported into ImageJ v1.53 (Schindelin et al., 2012) for analysis. The ‘segmented line’ tool was used to apply points along the head to tail axis of the body, and the ‘fit spline’ procedure was used to convert the line connecting these points to a smooth curve, which was taken to represent the midline of the animal. Stripes were counted from the nape of the neck to the tip of the tail, and were only considered as a full stripe if they bisected the midline and extended at least 50% of the torso width each side of the midline [Murray et al. (1990) counted stripes from the crown of the head to the tip of the tail, but because head and trunk pigmentation likely has distinct embryological origins in cranial and trunk neural crest, we treat these two areas differently]. ‘Torso’ stripes [those between the nape of the neck and the rump (where the back of the legs meets the body)] were recorded separately from ‘tail’ stripes (those between the rump and tail tip). Tail tip colour was also noted. Our final dataset comprised stripe counts from 279 individuals across four treatments (70 from each of the 29.5°C treatments, 73 from the 33.5°C+E2 treatment, and 66 from the 33.5°C+control treatment). The ‘plot profile’ tool of ImageJ was used to calculate average image pixel intensity across the widest part of the head for 158 hatchling images where the view of the dorsal surface of the head was uninterrupted, after white balance correction versus the white panel of the reference colour standard (https://github.com/pmascalchi/ImageJ_Auto-white-balance-correction/releases/tag/v1.0). Investigators were blinded to treatment during photographic analysis and data collection to remove unconscious test bias, and images were analysed in a randomized order.

### Statistical analysis

All statistical analyses were conducted in R version 4.1.2 (https://www.r-project.org/). Raw data are provided in Dataset 1. To examine the influence of incubation temperature, hormone treatment and their interaction on total length, body mass, stripe patterns and head coloration, linear mixed-effects models (LMMs) or generalized linear mixed-effects models (GLMMs) were fit with the R package ‘lme4’ (version 1.1-30; Bates et al., 2015). For each hatchling trait, the global model included fixed effects of incubation temperature, hormone treatment and their interaction. By testing for an interaction between temperature and hormone treatment, we were able to detect any influence of hatchling sex on hatchling traits. Clutch identity was also included as a random effect in all models to account for genetic relatedness among hatchlings. For the LMMs, the global model and reduced models were compared using likelihood-ratio tests to determine which terms significantly improved model fit. Terms that did not significantly improve model fit were subsequently removed (according to a significance threshold of *P*<0.05). The *P*-values for variables included in the final LMMs were derived from the ‘lmerTest’ package (version 3.1-3; Kuznetsova et al., 2017) using Satterthwaite's degrees of freedom method. For GLMMs, simulated likelihood-ratio tests based on non-parametric bootstrapping (*n*=500 simulations) in the package ‘DHARMa’ (version 0.4.6; https://cran.r-project.org/web/packages/DHARMa/DHARMa.pdf) were used to assess which terms significantly improved model fit. To assess the fit of the global model and diagnose deviations from model assumptions (e.g. over- or under-dispersion), we used the function simulateResiduals() within the package ‘DHARMa’ (version 0.4.6). Total length, body mass and head colour intensity were modelled using LMMs. Total stripes, torso stripes and tail stripes were initially modelled using GLMMs with a Poisson distribution; however, the resulting models showed evidence of under-dispersion. We subsequently modelled the binary response variable of a hatchling having a stripe count above the mean using GLMMs with a binomial distribution and logit link function. Tail tip colour was also modelled using a GLMM with a binomial distribution and logit link function.

## RESULTS AND DISCUSSION

**Fig. 1.**
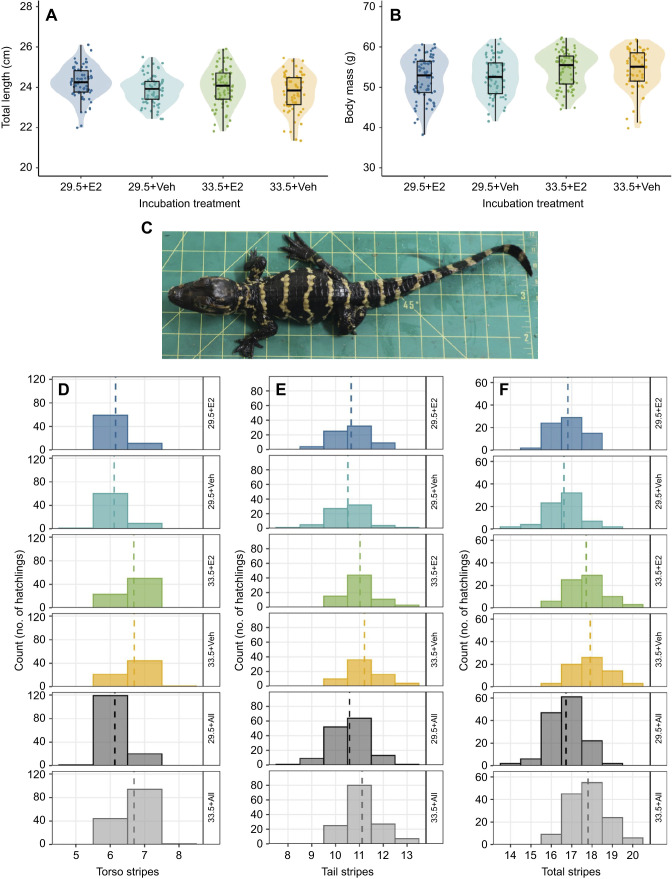
**Incubation temperature significantly affects hatchling length, mass and stripe number.** (A) Hatchling length, (B) mass and (D–F) stripe number. (C) The dorsal stripe pattern of a representative hatchling. Eggs were incubated at low (29.5°C) or high (33.5°C) temperature with either 17β-estradiol (E2) or control (vehicle-only, Veh) treatment. (A) Length was significantly affected by hormone treatment, with E2-treated hatchlings being longer than hatchlings treated with vehicle (24.13±0.07 versus 23.82± 0.07 cm, respectively). (B) Mass was significantly affected by incubation temperature, but not hormone treatment or the interaction of temperature and hormone treatment, with hatchlings incubated at 33.5°C having a greater body mass (mean±s.e.m.=54.47±0.4 g) than those incubated at 29.5°C (52.46±0.43 g). (D,E) Hatchlings incubated at the higher temperature tended to have more torso (mean±s.e.m.=6.69±0.04) and tail (11.12±0.06) stripes compared with hatchlings incubated at the lower temperature (D, torso stripes: 6.14±0.03; E, tail stripes: 10.59±0.07). (F) Animals incubated at the lower temperature had on average 16.72±0.07 total stripes whereas those incubated at the higher temperature had on average 17.81±0.08 total stripes. Data based on 279 individuals: 70 from each of the 29.5°C treatments; 73 from the 33.5°C+E2 treatment; and 66 from the 33.5°C+ vehicle treatment.

There was a significant effect of incubation temperature, but not hormone treatment or the interaction of temperature and hormone treatment, on hatchling body mass (estimate±s.e.=1.457±0.323, *P*<0.0001). Hatchlings incubated at the higher temperature (33.5°C) had a greater body mass (mean±s.e.m.=54.47±0.4 g) compared with hatchlings incubated at the lower temperature (29.5°C; 52.46± 0.43 g; Fig. 1). Variation in total length was significantly associated with both incubation temperature (estimate=−0.230± 0.079, *P*=0.004) and hormone treatment (estimate=0.343±0.079, *P*<0.0001), but not their interaction. Hatchlings incubated at the higher temperature were shorter (mean±s.e.m.=23.9±0.08 cm) compared with hatchlings incubated at the lower temperature (24.06±0.06 cm), and hatchlings treated with E2 as embryos were longer (24.13±0.07 cm) compared with hatchlings treated with vehicle (23.82±0.07 cm; Fig. 1). Total length and body mass were strongly correlated for all four incubation conditions (Spearman's rank correlation: *r*_277_=0.630, *P*<0.001).

Incubation temperature significantly influenced the number of torso stripes (estimate=3.019±0.3664, *P*<0.0001) and tail stripes (estimate=1.288, *P*<0.0001). Hatchlings incubated at the higher temperature tended to have a greater number of both torso stripes (mean±s.e.m.=6.69±0.04) and tail stripes (11.12±0.06) compared with hatchlings incubated at the lower temperature (torso stripes: 6.14±0.03; tail stripes: 10.59±0.07) (Fig. 1). Torso stripe and tail stripe patterns were unaffected by hormone treatment or the interaction of incubation temperature and hormone treatment. Overall, hatchlings incubated at 29.5°C had on average 16.72±0.07 total stripes whereas those incubated at 33.5°C had on average 17.81±0.08 total stripes (Fig. 1). This corresponded to a significant effect of incubation temperature on total stripe number (estimate=2.179±0.304, *P*<0.0001). In contrast, there were no significant effects of hormone treatment or the interaction of temperature and hormone treatment on total stripe number. Therefore, temperature significantly affected the number of dorsal stripes on *A. mississippiensis* hatchlings (*P*<0.001), with hatchlings incubated at 33.5°C having on average 1.09 stripes more than those incubated at 29.5°C (17.81 versus 16.72 stripes, respectively), and no significant difference in stripe number between the hormone treatment or vehicle-only control at either temperature. Our larger sample size and E2-induced sex reversal data agree with the findings of Murray et al. (1990), who proposed that this was related to embryo size at the pattern-forming stage of development, where those incubated at higher temperatures are larger and so can accommodate more stripes.

Whilst our hatchlings incubated at low and high temperatures differed by on average 1.09 stripes, the hatchlings incubated at 33°C by Murray et al. (1990) differed from those incubated at 30°C by 1.8 stripes (20.35 versus 18.55 stripes, respectively), which is explained by the fact that their hatchlings were overall longer than ours: 26.51 versus 23.91 cm for those incubated at low temperatures, and 27.00 versus 24.06 cm at high temperatures. Factors affecting hatchling morphology include genetics, resource availability, stress, egg size and mother size (Milnes et al., 2001; Stahlschmidt et al., 2013; Cameron et al., 2016), and the animals from the Murray et al. (1990) study were collected from Louisiana in the late 1980s, whereas ours were collected from South Carolina in 2020. There is obviously variation in stripe number within and between alligator populations, and it will be interesting to investigate the extent of this variation in hatchling phenotype.

We follow Murray et al. (1990) in differentiating hatchlings with white tail tips and those with black tail tips. Temperature had a significant effect on tail tip colour (estimate=0.889±0.251, *P*<0.0001), where 35.00% of hatchlings incubated at 29.5°C and 56.12% of hatchlings incubated at 33.5°C had a white tail tip. There was no significant effect of hormone treatment or the interaction of temperature and hormone treatment on tail tip colour. If a white tail tip is considered as an additional stripe, this would increase the difference in total stripe number between hatchlings from different incubation temperatures, with those incubated at the higher temperature having, on average, 1.3 more stripes than those incubated at the low temperature (18.37 versus 17.07 stripes, respectively). Although it is possible that the significant effect of incubation temperature on tail tipe colour stems from some unique development quirk of the tail bud (Gont et al., 1993; Tucker and Slack, 1995; Wymeersch et al., 2021), it seems more likely that this again supports the size-dependent pattern formation model and reflects a difference in size between embryos incubated at low and high temperatures. If stripes are formed by a wavefront that moves from head to tail, with peaks and troughs (Murray et al., 1990), then in some individuals the tail tip might correspond to a peak, and in others to a trough. Because distance between stripes is related to embryo length at the time of initiation of the pattern formation process, experimental groups in different incubation temperatures will have a greater or lesser proportion of individuals whose tail tip overlaps with the stripe-forming region.

**Fig. 2.**
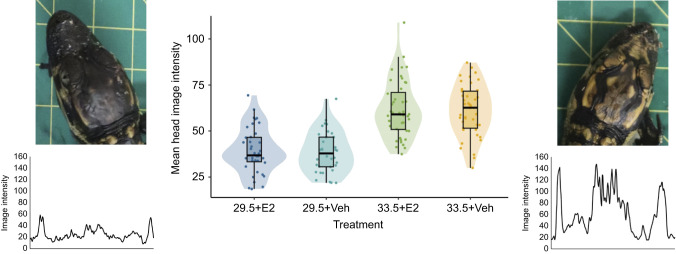
**Alligators incubated at the lower temperature (29.5°C) have significantly darker heads than those incubated at the higher temperature (33.5°C).** The centre graph shows average image intensity across the widest point of the head for each experimental condition [incubation temperature, plus treatment with E2 or vehicle (Veh)], and the side panels show representative individuals from each incubation temperature (left, 29.5°C; right, 33.5°C) and corresponding image intensity plots. Data based on 158 individuals for which head intensity measurements were possible: 39 from 29.5°C+E2 (mean image intensity=39.33±12.27), 24 from 29.5°C+Veh (mean=38.45±11.19), 44 from 33.5°C+E2 (mean=61.75±15.29) and 41 from 33.5°C+Veh (mean=61.16±14.06).

Finally, incubation temperature significantly influenced head colour intensity (estimate=22.691±2.055, *P*<0.0001). There was no statistically significant effect of hormone treatment or the interaction between the effects of incubation temperature and hormone treatment on head colour intensity. The mean head colour intensity of hatchlings from 33.5°C was ∼58% greater than that of hatchlings from 29.5°C (Fig. 2). The effect of temperature on this aspect of alligator hatchling morphology has, to our knowledge, not previously been identified, although it should perhaps not be too surprising, and has previously been reported for turtles (Deeming and Ferguson, 1991). The pigment cells of the head are also neural crest derivatives, although they arise from a distinct cell population: the cranial neural crest. Pigment cells of the trunk and tail are derivatives of the trunk neural crest, and these two populations differ in several ways, but perhaps most obviously by lack of an association or interaction with somites (Ferguson and Graham, 2004; Kuratani et al., 2018; Rocha et al., 2020). The stripe-forming model of Murray et al. (1990) begins only at the nape of the neck and extends along the length of the body to the tip of the tail, so the mechanisms underlying pigmentation patterning of the alligator head are currently unknown, but are clearly affected by incubation temperature.

Increased melanization such as this may have implications for thermoregulation in this ectothermic species, especially if replicated across the body surface and maintained throughout the lifespan. The thermal melanism hypothesis suggests that darker individuals have an advantage in colder climates (Clusella-Trullas et al., 2007), and has been investigated in a range of snake and lizard species (e.g. Andren and Nilson, 1981; Clusella-Trullas et al., 2009; Geen and Johnston, 2014; Reguera et al., 2014; Martínez-Freiría et al., 2020). Our results suggest that lower incubation temperature may be an unappreciated source of pigmentation variation in reptiles, with implications for the thermal melanism hypothesis.

In summary, our data show that incubation temperature affects stripe number, tail tip colour and head colouration in American alligators, and that this process is unaffected by E2-induced sex reversal. Early-life survival is tightly linked to predation risk by visual predators (e.g. birds), thus hatchling coloration likely serves as a critical crypsis mechanism in and around grasses (Somaweera et al., 2013). Given that these phenomena are temperature-related, impacts on coloration patterns may prove to be an unforeseen consequence of climate change for this species.

## Supplementary Material

10.1242/jexbio.245219_sup1 Supplementary information
