# Supplementary material for: Incubation temperature alters stripe formation and head colouration in American alligator hatchlings and is unaffected by estradiol-induced sex reversal
Source: J Exp Biol. 2023 Mar 31;226(6):jeb245219. doi: 10.1242/jeb.245219 (PMC10112970; doi:10.1242/jeb.245219)
Supplement: Supplementary information [file jexbio-226-245219-s1.pdf]

Dataset 1. Alligator data tables

Click here to download Dataset 1

Journal of Experimental Biology • Supplementary information
